# Genetic Ancestry and Asthma and Rhinitis Occurrence in Hispanic Children: Findings from the Southern California Children’s Health Study

**DOI:** 10.1371/journal.pone.0135384

**Published:** 2015-08-11

**Authors:** Muhammad T. Salam, Tigran Avoundjian, Wendy M. Knight, Frank D. Gilliland

**Affiliations:** 1 Department of Preventive Medicine, University of Southern California, Keck School of-Medicine, Los Angeles, California, United States of America; 2 Department of Psychiatry, Kern Medical Center, Bakersfield, California, United States of America; 3 US Department of Veterans Affairs, Center for Health Care Evaluation, Palo Alto, California, United States of America; 4 Los Angeles County Department of Public Health, Acute Communicable Disease Control Program, Los Angeles, California, United States of America; Universitat Pompeu Fabra, SPAIN

## Abstract

**Background:**

Asthma and rhinitis are common childhood health conditions. Being an understudied and rapidly growing population in the US, Hispanic children have a varying risk for these conditions that may result from sociocultural (including acculturative factors), exposure and genetic diversities. Hispanic populations have varying contributions from European, Amerindian and African ancestries. While previous literature separately reported associations between genetic ancestry and acculturation factors with asthma, whether Amerindian ancestry and acculturative factors have independent associations with development of early-life asthma and rhinitis in Hispanic children remains unknown. We hypothesized that genetic ancestry is an important determinant of early-life asthma and rhinitis occurrence in Hispanic children independent of sociodemographic, acculturation and environmental factors.

**Methods:**

Subjects were Hispanic children (5–7 years) who participated in the southern California Children’s Health Study. Data from birth certificates and questionnaire provided information on acculturation, sociodemographic and environmental factors. Genetic ancestries (Amerindian, European, African and Asian) were estimated based on 233 ancestry informative markers. Asthma was defined by parental report of doctor-diagnosed asthma. Rhinitis was defined by parental report of a history of chronic sneezing or runny or blocked nose without a cold or flu. Sample sizes were 1,719 and 1,788 for investigating the role of genetic ancestry on asthma and rhinitis, respectively.

**Results:**

Children had major contributions from Amerindian and European ancestries. After accounting for potential confounders, per 25% increase in Amerindian ancestry was associated with 17.6% (95% confidence interval [CI]: 0.74–0.99) and 13.6% (95% CI: 0.79–0.98) lower odds of asthma and rhinitis, respectively. Acculturation was not associated with either outcome.

**Conclusions:**

Earlier work documented that Hispanic children with significant contribution from African ancestry are at increased asthma risk; however, in Hispanic children who have little contribution from African ancestry, Amerindian ancestry was independently associated with lower odds for development of early-childhood asthma and rhinitis.

## Introduction

Chronic airway diseases including asthma and rhinitis are common health conditions in children [1–3]. In 2007, 13.1% of US children had been diagnosed as having asthma during their lifetime [1]. The annual prevalence for rhinitis in children in the United States ranges from 10–40% [2, 4]. An accumulating body of evidence indicates that genetic and environmental exposures during early-life may play particularly important roles in early-life development of asthma and rhinitis. Because young children have relatively high prevalence of asthma and rhinitis, identification of risk factors for these outcomes has received much attention in the last several decades.

In the United States, the Hispanic population has experienced the fastest growth than any other racial/ethnic group [5–6]. According to the 2010 US Census, approximately 16% of the US population is Hispanic and there was a 43% increase in this population group between 2000 and 2010 [6]. Hispanic children are a heterogeneous group with diverse cultural, socioeconomic and ancestral backgrounds, and have varying rates of asthma and rhinitis across ethnic groups. National surveillance data show that prevalence rates for asthma and allergy are the higher in Puerto Rican children than other Hispanic children [7–9]. Among Hispanic children of Mexican descent, studies have documented that children born in the US were at significant higher risk of asthma and allergy compared to those who were born in Mexico [10–13] suggesting a role of acculturation in allergic airway diseases. While it is not fully understood how acculturation may impact risk of atopic diseases, there are several putative mechanisms that may underlie the association of acculturation with allergic airway disease in Hispanic children. One such mechanism can be explained by the Hygiene Hypothesis which posits that in utero and early-life exposures to microbial exposure protects against atopic diseases [14]. Most newly immigrant Hispanic mothers and infants are likely to be exposed to higher level of microbial exposures in their country of origin compared those living in the US [13], which may reduce risk of developing atopic diseases such as asthma and rhinitis. In addition, lower prevalence of exposure to irritant exposures such as tobacco smoke from less acculturative Hispanic mothers [15–16] and higher consumption of fruits and vegetables in children from less acculturative background with higher serum antioxidant levels [17–18] may also explain lower prevalence of atopic disease with low acculturation.

To date, some studies have been conducted in an attempt to understand the reasons for the considerable differences in the prevalence rates of asthma and allergy between these different Hispanic groups. Puerto Rican children have significant contributions from African ancestry, which has been associated with higher asthma and allergy occurrences [19–20]. However, children of Mexican descent, who comprises the largest sub-group among Hispanics, have little contribution from African ancestry. This group has significant contribution of Amerindian ancestry, and whether this ancestry affects asthma and rhinitis risk is less investigated. One recent study conducted in older children and adults (age range 8 to 40) documented lower odds of developing asthma with higher proportion of Amerindian ancestry [21]; however, whether acculturative factors influenced this association was not reported. The impact of genetic ancestry on rhinitis risk remains unknown.

In the present study, we aimed to investigate the joint associations of genetic ancestry, acculturation, sociodemographic factors and environmental exposures on early-childhood asthma and rhinitis occurrence by age 7 in a well characterized population of Hispanic children who were born in California and participated in the southern California Children’s Health Study (CHS).

## Materials and Methods

### Study design and subjects

The study population consisted of Hispanic children from a cohort of kindergarten and first grade students (age 5–7 years) who were recruited in 2003 from thirteen communities as part of the CHS. Details of the study population recruitment methods have been presented previously [22]. Birth certificate information for California-born children was obtained by computerized linkage of participants with the California Department of Health Services Birth Statistical Master Files and Birth Cohort Files. Buccal samples were collected for genotyping. The institutional review board at the University of Southern California approved the study protocol and parents or legal guardians provided written informed consents.

**Fig 1:**
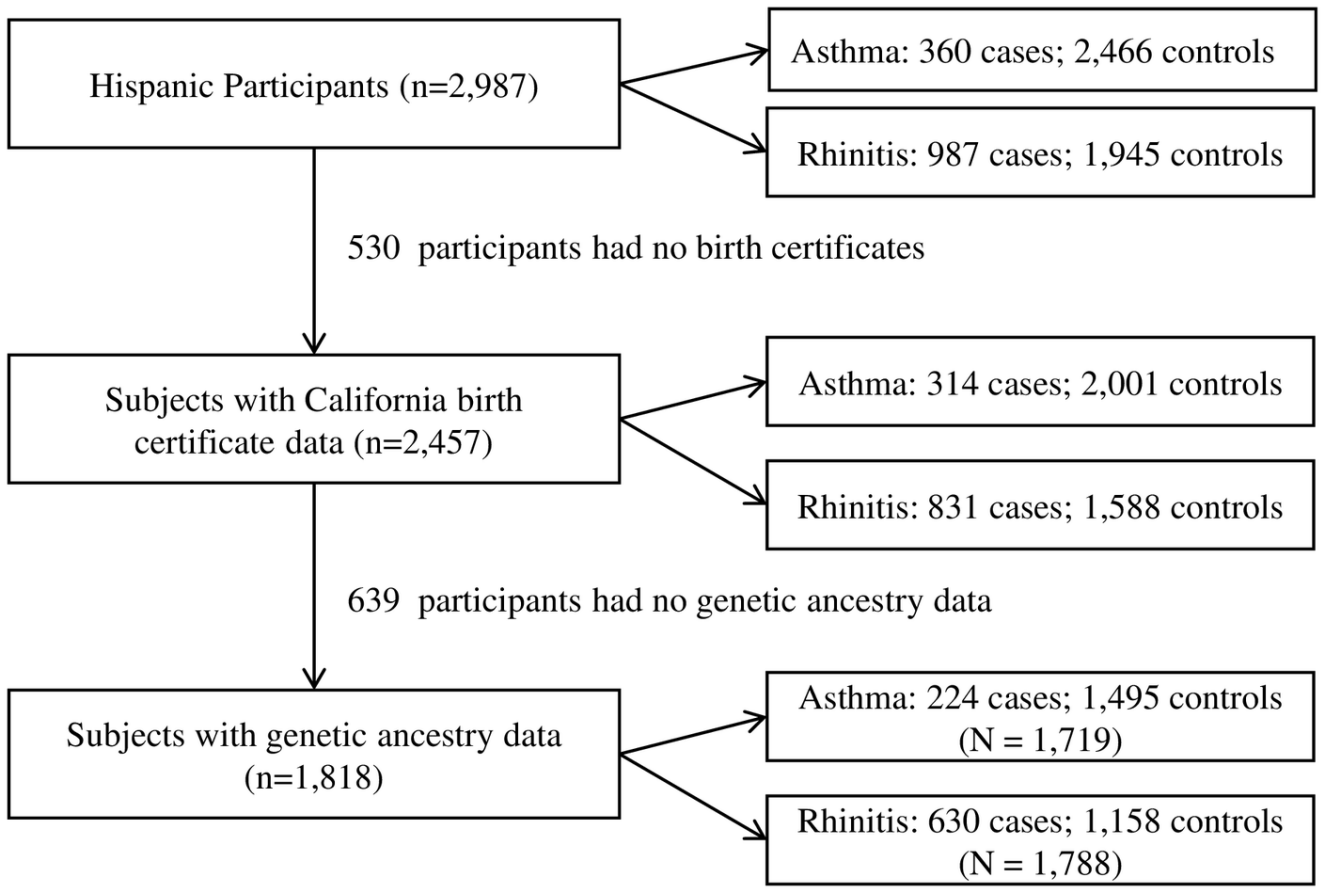
Flow Chart of Study Population and Sample Size. The figure shows the number of Hispanic children available in the Children’s Health Study in 2003, subjects excluded for not having birth certificate or genetic ancestry data, and final sample size available for analyzing the associations of genetic ancestry on asthma (N = 1,719) and rhinitis (N = 1,788).

Ethnicity was defined by parental report of race and whether they considered their child to be Hispanic. There were 2,987 Hispanic children at study entry (Fig 1). Because data on maternal birthplace was available from California birth certificates, we excluded 530 children without birth certificate data. An additional 639 subjects were excluded because buccal samples and/or genetic ancestry data were unavailable. Our final sample sizes were 1,719 and 1,788 Hispanic children for investigating the role of ancestry, acculturation, sociodemographic factors and environmental exposures on asthma and rhinitis, respectively.

### Outcomes definitions

Asthma was defined based on parental response to the question "Has a doctor ever diagnosed this child as having asthma?" Rhinitis was defined based on a positive response to the question, "has your child ever had a problem with sneezing or a runny or blocked nose, when he/she did not have a cold or the ‘flu?" Diagnosis of asthma and rhinitis was based on parental report. Based on medical records review, we have previously found strong evidence that parental report reflected physician diagnosis of asthma [23]. While there is no gold-standard definition for rhinitis, we have employed the methods used in the International Study of Asthma and Allergies in Childhood (ISAAC) questionnaire in this study [24]. Furthermore, the symptoms of nasal congestion, runny nose and sneeze were found to be the most common complaints in children with rhinitis as reported by them, their parents and healthcare providers [2]. We used ISAAC question "Have your child ever had a problem with sneezing, or a runny, or blocked nose when he/she DID NOT have a cold or the flu?" to define rhinitis status. We did not have data on skin prick testing or serum immunoglobulin E to objectively define atopy because subjects were recruited from public school classrooms and such invasive testing was not feasible.

### Determination of genetic ancestry

We followed the methods utilized by Smith et al [25] to select ancestry informative markers (AIMs) to estimate genetic ancestry. In brief, Smith et al selected 400 AIMs spaced by at least 25cM from a validated list of 3,011 AIMs, with 100 AIMs each to optimally distinguish between European-West African, European-Amerindian, European-East Asian and between West African-Amerindian ancestral origins and to make precise ancestry estimates. The average differences in allele frequencies were 78% for African/European mixture, 85% for African/Amerindian mixture, 56% for European/Amerindian mixture and 57% for European/East-Asian mixture. We picked 233 AIMs from the list of 400 AIMs that are unique and had a high probability of successful genotyping using Illumina GoldenGate assay.

These AIMs were genotyped using the Illumina BeadArray platform. All SNPs had call rates >99%. We used the STRUCTURE program (available at http://pritchardlab.stanford.edu/structure.html) to differentiate the four major ancestral populations [26]. When running STRUCTURE, the number of clusters (K) is predefined to a range of numbers based on our knowledge about the samples under studying, and the admixture model is used for running the program [27]. The length of burnin period was set at 20,000, and the number of MCMC steps after burnin was set at 10,000. For each K, 10 independent runs were performed and the final number of clusters K used to interpret the sub-structures was decided based on the estimated Ln probability of the data. We used a four population model because it was found to be the most probable given the data.

### Determination of acculturation

Information on maternal birthplace was available on birth certificates, and children were grouped into 3 categories (i.e., mothers born in the United States, Mexico, or elsewhere). At study entry, parents completed the questionnaire either in English or in Spanish. We used this information to assess parents’ language preference. Based on maternal birthplace and parental language preference, we formulated a composite variable with six categories to assess acculturation.

### Assessment of covariates

Race/ethnicity, annual family income, parental education, child's health insurance coverage, parental history of asthma/allergy, preterm birth, exposure to *in utero* and secondhand tobacco smoke (SHS) and presence of cat, dog and cockroach at home were based on parental reports at study entry. Data on birth weight, preterm birth and mode of delivery were available from the birth certificates. Height and weight were measured by study personnel at schools. Age- and sex-specific percentiles based on the Centers for Disease Control and Prevention body mass index (BMI) growth charts were used to categorize BMI into underweight, normal, overweight and obese. We estimated distance of each participant’s residence to the nearest major road and categorized it as <75m, 75-150m, 151-300m, and > 300m based on a previous paper which reported significant increased risk of asthma in the overall population of CHS children who lived near a major road [22].

### Statistical analyses

We described the population characteristics by health outcomes and investigated the univariate relationships between risk factors and asthma and rhinitis using independent t-tests or Mann-Whitney U tests for continuous variables and Pearson’s chi-square tests for categorical variables. We also evaluated the relationships between sociodemographic factors and environmental exposures and Amerindian ancestry. Finally, logistic regression was used to evaluate the independent and joint associations of Amerindian ancestry and acculturation with asthma and rhinitis after accounting for potential confounders. Potential confounding covariates were included in final models if their inclusion resulted in a 10% change in the odds ratio for ancestry on the outcomes. We examined the modifying effects of gender, parental history of asthma/allergy, annual family income, exposure to tobacco smoke in utero or during childhood, exposures to cat, dog or cockroach at home or by residential distance to the nearest major road on the relationships between Amerindian ancestry and asthma and rhinitis with incorporation of appropriate interaction terms in the final models. All tests were two-sided at a 5% significance level. All statistical analyses were performed using SAS software v. 9.2 (SAS Institute, Inc., Cary, NC, USA).

## Results

Asthma and rhinitis prevalences were 13.03% and 35.23%, respectively (Table 1). In univariate analyses, male gender and parental history of asthma/allergy were associated with higher odds of asthma and rhinitis, whereas children born to mothers who were born in Mexico or whose parents completed the questionnaire in Spanish had significantly lower odds. Maternal smoking during pregnancy was significantly associated with higher odds of rhinitis and in utero exposure to paternal smoking was associated with lower odds of asthma and rhinitis. Preterm birth and presence of cat at home were associated with higher odds of rhinitis but not with asthma. Subjects included in the present study were more likely to have health insurance and less likely to have family income <$15,000 and exposures to tobacco smoke and cockroach (Table 2).

**Table 1:** Characteristics of California-born Hispanic Participants in Children’s Health Study by Asthma and Rhinitis Status.^a^

Subjects’ characteristics	Asthma			Rhinitis		
	Controls (N = 1,495)	Cases (N = 224)	*P* value^c^	Controls (N = 1,158)	Cases (N = 630)	*P* value^c^
	N^b^ (%)	N^b^ (%)		N^b^ (%)	N^b^ (%)	
Age (years)^d^	5.99 (0.74)	5.99 (0.79)	.91	5.98 (0.74)	6.01 (0.76)	.47
Boys	733 (49.03)	137 (61.16)	**< .001**	559 (48.27)	350 (55.56)	**.003**
Annual family income						
<$15,000	258 (20.36)	30 (15.87)		202 (20.74)	107 (19.71)	
$15,000–$49,999	576 (45.46)	81 (42.86)	.12	443 (45.48)	237 (43.65)	.53
≥$50,000	433 (34.18)	78 (41.27)		329 (33.78)	199 (36.65)	
Maternal birth place						
United States	672 (44.95)	144 (64.29)		497 (42.92)	354 (56.19)	
Mexico	749 (50.10)	70 (31.25)	**< .001**	604 (52.16)	244 (38.73)	**< .001**
Other	74 (4.95)	10 (4.46)		57 (4.92)	32 (5.08)	
Birth by Cesarean section	278 (18.60)	45 (20.09)	.59	212 (18.31)	125 (19.84)	.43
Preterm birth	131 (8.97)	26 (11.71)	.19	93 (8.18)	74 (12.09)	**.008**
Low birth weight (<2500g)	74 (4.95)	11 (4.91)	.98	57 (4.92)	33 (5.24)	.77
Parent completed Spanish questionnaire	584 (39.06)	57 (25.45)	**< .001**	463 (39.98)	208 (33.02)	**.004**
Child has health insurance	1242 (86.31)	203 (91.86)	**.02**	957 (85.68)	554 (90.08)	**.009**
Any parental history of asthma/allergy	495 (36.32)	130 (65.33)	**< .001**	310 (29.27)	344 (61.10)	**< .001**
Exposed to maternal smoking in utero	53 (3.64)	10 (4.63)	.47	34 (3.01)	38 (6.22)	**.001**
Exposed to paternal smoking in utero	176 (12.26)	39 (18.31)	**.01**	129 (11.58)	97 (16.14)	**.008**
Children exposed to second hand smoke	93 (6.38)	22 (10.00)	.05	81 (7.14)	43 (6.95)	.86
Residential distance from a major road						
<75m	506 (38.10)	84 (40.98)		393 (38.34)	228 (39.86)	
75-150m	355 (26.73)	43 (20.98)	.38	278 (27.12)	135 (23.60)	.40
151-300m	246 (18.52)	41 (20.00)		182 (17.76)	114 (19.93)	
>300m	221 (16.64)	37 (18.05)		172 (16.78)	95 (16.61)	
Presence of cat at home	169 (11.76)	32 (14.95)	.18	117 (10.50)	92 (15.18)	**.005**
Presence of dog at home	358 (24.91)	50 (22.36)	.62	269 (24.15)	159 (26.24)	.34
Presence of cockroach at home	168 (12.47)	18 (8.82)	.13	117 (11.37)	81 (13.94)	.13
BMI Category						
Underweight	65 (4.69)	6 (2.97)		56 (5.19)	22 (3.83)	
Normal	843 (60.78)	126 (62.38)	.55	646 (59.93)	368 (64.00)	.28
Overweight	227 (16.37)	29 (14.36)		173 (16.05)	91 (16.05)	
Obese	252 (18.17)	41 (20.30)		203 (18.83)	94 (16.35)	

^a^ Hispanic children in the Children’s Health Study with birth certificate and genetic ancestry data. For details, see the methods.

^b^ Numbers always do not add up because of missing data.

^c^ *P* values from Pearson chi-square tests for categorical variables and independent t-tests for age comparing asthma cases to controls and rhinitis cases to controls. Statistically significant P-values are in **bold**.

^d^ Mean (SD) are provided for age.

**Table 2:** Comparison of Hispanic children in Children's Health Study who are included in the present study vs. those who were not included.^a^

Subjects’ characteristics	Hispanic children in Children’s Health Study				P-value^c^
	Included in present analysis		Not included in present analysis		
	(N = 1,818)		(N = 1,169)		
	N^b^	(%)	N^b^	(%)	
Age (years)^d^	5.99	(0.74)	6.07	(0.81)	**.01**
Boys	925	(50.88)	592	(50.64)	.90
Annual family income					
<$15,000	315	(20.55)	314	(33.95)	**< .0001**
$15,000–$49,999	689	(44.94)	393	(42.49)	
≥$50,000	529	(34.51)	218	(23.57)	
Preterm birth	167	(9.41)	112	(9.91)	.65
Parent completed Spanish questionnaire	689	(37.90)	570	(48.76)	**< .0001**
Child has health insurance	1529	(87.12)	832	(74.95)	**< .0001**
Any parental history of asthma/allergy	659	(40.06)	308	(30.56)	**< .0001**
Exposed to maternal smoking in utero	73	(4.14)	60	(5.49)	.10
Exposed to paternal smoking in utero	228	(13.10)	192	(17.76)	**.0007**
Children exposed to second hand smoke	126	(7.12)	105	(9.36)	**.03**
Residential distance from a major road					
<75m	628	(38.72)	343	(36.61)	.50
75-150m	420	(25.89)	256	(27.32)	
151-300m	302	(18.62)	190	(20.28)	
>300m	272	(16.77)	148	(15.80)	
Presence of cat at home	212	(12.14)	117	(10.48)	.18
Presence of dog at home	433	(24.79)	245	(21.95)	.08
Presence of cockroach at home	202	(12.36)	195	(18.64)	**< .0001**
BMI Category					
Underweight	78	(4.64)	33	(3.24)	.30
Normal	1030	(61.31)	647	(63.56)	
Overweight	269	(16.01)	159	(15.62)	
Obese	303	(18.04)	179	(17.58)	
Children with asthma	224	(13.03)	136	(12.29)	.56
Children with rhinitis	630	(35.23)	357	(31.21)	**.02**

^a^Hispanic children for whom asthma and rhinitis information was available and on whom birth certificate and genetic ancestry data were available were included in the present study. Hispanic children who lacked these data were who were excluded for this study.

^b^ Numbers always do not add up because of missing data.

^c^ *P* values from Pearson chi-square tests for categorical variables and independent t-tests for age comparing Hispanic children who are included in the present analysis to those who are not included in the analysis (see methods for details regarding reasons for exclusions of subjects from the present analysis). Statistically significant P-values are in **bold**.

^d^ Mean (SD) are provided for age.

The major ancestral contributions were from Amerindian and European ancestries with little contributions from African and Asian ancestries (Table 3). In univariate analysis, children with asthma and rhinitis had lower proportion of Amerindian ancestry and higher proportion of European ancestry compared to unaffected children. For 25% increase in African ancestry, there was 51% higher odds of asthma; however, the odds ratio was of borderline statistical significance (P = 0.05).

**Table 3:** Distributions of Genetic Ancestry and Univariate relationship between Genetic Ancestry and Asthma and Rhinitis.

Genetic ancestry	Asthma			Rhinitis		
	Controls Median (IQR)	Cases Median (IQR)	OR (95% CI)^a^	Controls Median (IQR)	Cases Median (IQR)	OR (95% CI)^a^
European	0.19 (0.47)	0.34 (0.59)	Ref.	0.18 (0.45)	0.29 (0.58)	Ref.
Amerindian	0.74 (0.51)	0.56 (0.63)	**0.77 (0.69–0.86)**	0.75 (0.48)	0.65 (0.62)	**0.83 (0.76–0.90)**
African	0.02 (0.03)	0.02 (0.04)	1.51 (1.00–2.30)	0.01 (0.03)	0.02 (0.03)	1.28 (0.87–1.90)
Asian	0.01 (0.01)	0.01 (0.02)	1.15 (0.73–1.82)	0.01 (0.01)	0.01 (0.01)	0.94 (0.65–1.36)

^a^ Unadjusted odds ratios (ORs) are compared to European ancestry and are scaled to 25% increases in Amerindian, African and Asian ancestries. Statistically significant ORs are in **bold**.

IQR, interquartile range.

Disease prevalence rates and distributions of sociodemographic and environmental exposures showed marked variation in Hispanic children having varying proportions of Amerindian ancestry (Table 4). Children with higher Amerindian ancestral proportion were more likely to come from a low socioeconomic status (SES; reflected by annual family income and child's health insurance status). They were also more likely to have mothers who were born in Mexico and have parents who preferred Spanish to complete the questionnaire. For environmental exposures, children with higher proportions of Amerindian ancestry were more likely to have exposure to cockroach at home and lived closer to a major road, but were less likely to have *in utero* exposure to parental smoking and pets (cats and dogs).

**Table 4:** Associations between Participants’ Sociodemographic Characteristics and Exposures with Amerindian Ancestry.

	Categories based on proportions of Amerindian Ancestry				P-value^a^
	<0.25	≥0.25 and <0.50	≥0.50 and <0.75	≥ 0.75	
	(N = 311)	(N = 245)	(N = 409)	(N = 853)	
	N (%)	N (%)	N (%)	N (%)	
Boys	165 (53.05)	115 (46.94)	221 (54.03)	424 (49.71)	.24
Children with asthma	61 (20.61)	38 (16.67)	53 (13.52)	72 (8.97)	**< .001**
Children with rhinitis	136 (43.87)	104 (42.98)	138 (34.24)	252 (30.25)	**< .001**
Annual family income					
<$15,000	26 (9.00)	25 (11.68)	60 (17.19)	204 (29.96)	
$15,000–$49,999	74 (25.61)	67 (31.31)	174 (49.86)	374 (54.92)	**< .001**
≥$50,000	189 (65.40)	122 (57.01)	115 (32.95)	103 (15.12)	
Maternal birth place					
United States	273 (87.78)	179 (73.06)	198 (48.41)	208 (24.38)	
Mexico	16 (5.14)	53 (21.63)	200 (48.90)	600 (70.34)	**< .001**
Other	22 (7.07)	13 (5.31)	11 (2.69)	45 (5.28)	
Birth by Cesarean section	65 (20.90)	65 (26.53)	77 (18.83)	137 (16.06)	**.002**
Preterm birth	33 (10.65)	31 (12.81)	43 (10.72)	60 (7.30)	**.03**
Low birth weight (<2500g)	9 (2.89)	14 (5.71)	23 (5.62)	46 (5.39)	.29
Parent completed Spanish questionnaire	9 (2.89)	34 (13.88)	142 (34.72)	504 (59.09)	**< .001**
Child has health Insurance	288 (94.12)	219 (91.63)	350 (86.63)	672 (83.37)	**< .001**
Any parental history of asthma/allergy	180 (61.86)	123 (53.71)	151 (40.70)	205 (27.19)	**< .001**
Exposed to maternal smoking in utero	37 (12.05)	10 (4.22)	13 (3.31)	13 (1.57)	**< .001**
Exposed to paternal smoking in utero	64 (21.40)	33 (13.92)	53 (13.52)	78 (9.61)	**< .001**
Children exposed to second hand smoke	23 (7.44)	18 (7.47)	34 (8.56)	51 (6.20)	.49
Residential distance from a major road					
<75m	137 (48.41)	109 (48.66)	129 (34.31)	253 (34.24)	
75-150m	63 (22.26)	40 (17.86)	106 (28.19)	211 (28.55)	**< .001**
151-300m	45 (15.90)	43 (19.20)	70 (18.62)	144 (19.49)	
>300m	38 (13.43)	32 (14.29)	71 (18.88)	131 (17.73)	
Presence of cat at home	84 (27.18)	56 (23.43)	31 (7.89)	41 (5.09)	**< .001**
Presence of dog at home	112 (36.25)	72 (30.13)	88 (22.39)	161 (19.98)	**< .001**
Presence of cockroach at home	15 (5.00)	16 (7.05)	38 (10.19)	133 (18.12)	**< .001**
BMI Category					
Underweight	26 (9.12)	6 (2.59)	14 (3.69)	32 (4.08)	
Normal	182 (63.86)	158 (68.10)	242 (63.85)	448 (57.14)	**< .001**
Overweight	42 (14.74)	39 (16.81)	59 (15.57)	129 (16.45)	
Obese	35 (12.28)	29 (12.50)	64 (16.89)	175 (22.32)	

^a^ P values from Pearson chi-square tests for overall association with categories of Amerindian ancestry. Statistically significant P-values are in **bold**.

Amerindian ancestry was associated with lower odds of asthma and rhinitis (Tables 5 and 6). After accounting for potential confounders, per 25% increase in Amerindian ancestry was associated with 17.6% (OR = 0.85; 95% CI: 0.74–0.99) and 13.6% (OR = 0.88; 95% CI: 0.79–0.98) lower odds of asthma and rhinitis in final models (Model 4), respectively. Because presence of cockroach was less likely in children’s home where a parent had asthma/allergy, adjustment for parental history in the final model showed that exposure to cockroach was associated with 47% higher odds of rhinitis (95% CI: 1.05, 2.05). For both outcomes, male gender and parental history of asthma/allergy were associated with higher odds.

**Table 5:** Multivariate Association between Amerindian Genetic Ancestry and Asthma.

	Model 1	Model 2	Model 3	Model 4
	OR (95% CI)^a^	OR (95% CI)^a^	OR (95% CI)^a^	OR (95% CI)^a^
Male gender	**1.64 (1.22–2.20)**	**1.62 (1.20–2.18)**	**1.62 (1.20–2.18)**	**1.61 (1.19–2.17)**
Any parental history of asthma/allergy	**3.19 (2.29–4.45)**	**2.97 (2.12–4.16)**	**2.87 (2.03–4.08)**	**2.87 (2.02–4.09)**
Annual family income				
<$15,000	1.02 (0.62–1.67)	1.15 (0.69–1.91)	1.15 (0.69–1.92)	1.18 (0.70–1.99)
$15,000–$49,999	1.04 (0.72–1.50)	1.18 (0.81–1.72)	1.15 (0.79–1.68)	1.21 (0.82–1.78)
≥$50,000	Ref	Ref	Ref	Ref
Amerindian ancestry^b^		**0.83 (0.72–0.95)**		**0.85 (0.74–0.99)**
Acculturation				
Mother Mexico born; prefers Spanish			0.71 (0.46–1.10)	0.90 (0.57–1.43)
Mother born elsewhere; prefers Spanish			0.33 (0.07–1.43)	0.40 (0.09–1.78)
Mother US born; prefers Spanish			1.36 (0.43–4.33)	1.72 (0.53–5.55)
Mother Mexico born; prefers English			**0.49 (0.29–0.84)**	0.60 (0.35–1.04)
Mother born elsewhere; prefers English			1.06 (0.46–2.32)	0.97 (0.43–2.21)
Mother US born; prefers English			Ref.	Ref.

^a^ Odds ratios (ORs) are adjusted for the variables with ORs shown under each model plus age and community of residence. Also models that included Amerindian ancestry (i.e., models 2 and 4) are further adjusted for African and Asian genetic ancestries. Statistically significant ORs are in **bold**.

^b^The OR for Amerindian ancestry is scaled to 25% increases in Amerindian ancestry.

**Table 6:** Multivariate Association between Amerindian Genetic Ancestry and Rhinitis.

	Model 1	Model 2	Model 3	Model 4
	OR (95% CI)^a^	OR (95% CI)^a^	OR (95% CI)^a^	OR (95% CI)^a^
Male gender	**1.33 (1.08–1.63)**	**1.32 (1.08–1.62)**	**1.33 (1.08–1.63)**	**1.33 (1.08–1.64)**
Any parental history of asthma/allergy	**3.94 (3.13–4.97)**	**3.79 (3.00–4.79)**	**3.92 (3.08–5.00)**	**3.91 (3.07–4.99)**
Cockroach exposure	**1.46 (1.05–2.04)**	**1.52 (1.09–2.13)**	**1.42 (1.02–1.99)**	**1.47 (1.05–2.05)**
Annual family income				
<$15,000	1.35 (0.96–1.90)	**1.48 (1.05–2.11**)	1.34 (0.94–1.91)	1.35 (0.96–1.90)
$15,000–$49,999	1.20 (0.92–1.57)	1.31 (0.99–1.74)	1.22 (0.93–1.62)	1.20 (0.92–1.57)
≥$50,000	Ref	Ref	Ref	Ref
Amerindian ancestry^b^		**0.88 (0.80–0.98)**		**0.88 (0.79–0.98)**
Acculturation				
Mother Mexico born; prefers Spanish			1.01 (0.75–1.37)	1.18 (0.85–1.62)
Mother born elsewhere; prefers Spanish			1.21 (0.58–2.50)	1.38 (0.66–2.88)
Mother US born; prefers Spanish			1.06 (0.42–2.65)	1.23 (0.49–3.12)
Mother Mexico born; prefers English			**0.63 (0.44–0.90)**	0.72 (0.50–1.04)
Mother born elsewhere; prefers English			0.80 (0.43–1.52)	0.79 (0.41–1.50)
Mother US born; prefers English			Ref.	Ref.

^a^ Odds ratios (ORs) are adjusted for the variables with ORs shown under each model plus age and community of residence. Also models that included Amerindian ancestry (i.e., models 2 and 4) are further adjusted for African and Asian genetic ancestries. Statistically significant ORs are in **bold**.

^b^The OR for Amerindian ancestry is scaled to 25% increases in Amerindian ancestry.

None of the associations presented in Tables 5 and 6 varied by gender, parental history of asthma/allergy, annual family income, exposure to tobacco smoke in utero or during childhood, exposures to cat, dog or cockroach at home or by residential distance to the nearest major road (all P-values for interaction > 0.20). Further adjustment for additional covariates presented in Table 1 did not substantially change the ORs. Finally, we conducted four sensitivity analyses for each outcome where we examined the relationships between Amerindian ancestry and asthma and rhinitis in (a) children who were born to US-born mothers, (b) children whose parents completed the study questionnaire in English, (c) in children whose annual family income was ≥$15,000, and (d) in children who had health insurance coverage. In all these sensitivity analyses, the ORs between Amerindian ancestry and asthma and rhinitis remained essentially unchanged (results not shown).

## Discussion

In Hispanic children who were born and lived in California, children with higher proportions of Amerindian ancestry had significantly lower odds of asthma and rhinitis development by age 7. Although sociocultural factors and environmental exposures were strongly related to Amerindian ancestry, the relationships between Amerindian ancestry and asthma and rhinitis remained robust after accounting for these factors. The established risk factors (male gender and parental history of asthma/allergy) were associated with higher odds of developing these two conditions; however, the relationships of Amerindian ancestry with asthma and rhinitis were independent of these factors. After accounting for ancestry and other covariates, acculturation was not significantly associated with odds of developing asthma or rhinitis.

Distributions of sociodemographic and asthmogenic environmental exposures vary significantly by genetic ancestry indicating that environmental justice issues affect some subgroups of Hispanic children more than others. While children with greater proportion of Amerindian ancestry had higher odds of being exposed to some risk factors (e.g., obesity, traffic-related pollution, exposure to cockroach), they were less likely to be exposed to tobacco smoke *in utero* and during early childhood and to pets (cats and dogs) at home. However, distributional difference of these factors did not account for the observed relationships between genetic ancestry and asthma and rhinitis.

Our findings may have public health implications as we showed variations in asthma and rhinitis prevalence rates in relation with Amerindian ancestry within a population that has often been considered as a single ethnic entity for disease surveillance purposes [1, 9, 28]. Further studies are warranted to determine genetic susceptibility for asthma and rhinitis in Hispanic children while incorporating genetic admixture information.

Although fewer children with higher proportions of Amerindian ancestry had a parental history of asthma/allergy (providing additional support of a protective effect on Amerindian ancestry on these conditions), we found that the protective effects of Amerindian ancestry on asthma and rhinitis did not vary by parental history of asthma/allergy. While a previous study documented that SES modified the relationship between African ancestry and asthma in Puerto Rican children [29], we did not find that the relationship between Amerindian ancestry and asthma and rhinitis varied by SES in our analyses.

This study had several advantages. The majority of published work to date has reported the role of African genetic ancestry on asthma and allergy in populations that have higher proportion of African ancestry (e.g., African-Americans, Puerto Ricans, Caribbean Colombians, Brazilians, Jamaicans, and Barbadians) [29–32]. To the best of our knowledge, this is the first study that documented that Amerindian ancestry is associated with lower odds of developing asthma and rhinitis in young children with little contribution of African ancestry while accounting for proxy measures of acculturative factors. Earlier work had documented the role of genetic ancestry or acculturation on asthma and allergy; however, we evaluated both factors in our analyses in conjunction with other asthmogenic factors.

There are some study limitations that need to be acknowledged. The use of maternal birthplace and Spanish language preference are proxy measures of acculturation that may not fully represent the degree to which individual participants have been acculturated in the United States. However, the independent effect of Amerindian ancestry on the study outcomes were not influenced by acculturation as the relationships between ancestry and asthma and rhinitis remained unchanged in restricted analysis conducted among children whose mothers were born in the US or in those whose parents completed the study questionnaire in English. It is possible that lack of health insurance could influence diagnosis by a physician; however, the results remained unchanged in terms of direction and magnitude of associations when the analyses were restricted to children with health insurance.

We acknowledge that some of the subjects included in the analysis to investigate the relationship between genetic ancestry and asthma were also included in the paper by Pino-Yanes et al [21]; however, methodologies (study population and analytic strategy) differed between these two studies. First, rhinitis was not considered as an outcome in the paper by Pino-Yanes et al, and as such no overlaps exist between the studies. Second, we conducted our study among 5–7 year old children to investigate the independent associations of genetic ancestry, environmental exposures and proxies of acculturation with early-life development of asthma and rhinitis and mentioned this in the introduction section of the revised manuscript, whereas Pino-Yanes et al conducted their study among 8–40 year old. Third, asthma definition was different between the two studies. Of the 1,818 children who are included in the asthma analysis in the present study, 749 (41.2%) children were included in the replication phase in the Pino-Yanes et al paper. The rest of the sample in Pino-Yanes and colleagues' paper was from other earlier CHS cohorts of 4^th^ (average age 10 yrs) and 7^th^ (average age 12 years) grader students who were recruited in 1993 and 1996. In the paper by Pino-Yanes et al, asthma was defined as asthma diagnosed by age 18 for cohorts recruited in 1993 and 1996 and until 2007 for the cohort that was recruited in 2003. However, in the present study asthma is defined as physician diagnosis at cohort entry in Kindergartners and first-graders (age 5–7 years) since we were interested in etiology of early-onset asthma development. Due to this difference in asthma definition, 177 children who developed asthma in adolescence and defined as cases in the paper by Pino-Yanes et al. were considered as controls in our paper. Fourth, we have considered many environmental and acculturative factors for potential confounding and conducted our study among 5–7 years old children to investigate the influence of genetic ancestry and acculturative factors on early-life asthma and rhinitis occurrence, whereas Pino-Yanes et al did not investigate the impact of acculturative factors on this association. Finally, we found that children in the CHS who had higher proportion of Amerindian ancestry were more likely to be lost in follow-up years and they were more likely to have low socioeconomic background (as reflected by family annual income, parental education and child's insurance status, data not shown). We therefore limited our analyses to data collected at cohort entry to investigate associations of genetic ancestry on early-life asthma and rhinitis occurrence and to limit introduction of any possible bias by incorporating data from follow-up years. So, while there is some overlap in subjects for the asthma analysis, the methodologies are different. Given the significant interrelationships of environmental and acculturative factors on genetic ancestry, we were able to conduct a thorough analysis of the genetic ancestry effects on asthma and rhinitis in 5–7 year old children after accounting for environmental and acculturation factors.

Many acculturation scales are available for Hispanic populations [33–35] and these were reviewed by Thomson and Hoffman-Goetz [36]. The majority of these scales include language preference and generation status in measuring acculturation in Hispanic populations. While we did not include any full acculturation scale in our study, we used two proxy measures of language used to complete the questionnaire and maternal birthplace to capture language preference and generation status. While we acknowledge that any measurement of acculturation with respect to dynamic changes in social, cultural, psychological, behavioral norms is too complex, proxy measures of acculturation have been used in many epidemiological studies for different health outcomes (reviewed by Thomson and Hoffman-Goetz [36]) including asthma in children [10].

Asthma diagnosis was based on parental report of physician diagnosis. While we previously found in our cohort that such report accurately represents physician diagnosis, it is plausible that parents of children with high Amerindian ancestry may decide not to go to the physician. That might affect asthma prevalence, and we acknowledge this as a study limitation. While asthma diagnosis relied on parental report of physician diagnosis, we relied on parental report of symptoms to define rhinitis as was done in ISAAC studies. Therefore, it is unlikely that our result for rhinitis is influenced by parental psychosocial behavior in seeking medical attention for their children. While parental psychosocial behavior in regards to seeking medical care for their children was not measured in our study, such behavior is likely to be related to maternal birthplace, socioeconomic status (as reflected by parental education and annual family income) and acculturation proxies. We have adjusted for these factors in our models, conducted several sensitivity analyses and observed similar direction of association with asthma and rhinitis.

There were some differences in subject characteristics between children who were included in the final analysis and those who were not included due to lack of birth certificate and/or genetic ancestry data. However, we have adjusted for these factors in our analyses and conducted sensitivity analyses within subgroups and found no substantial impact on the odds ratios.

In conclusion, we found that in young Hispanic children with little contribution from African ancestry, asthma and rhinitis prevalence rates vary by Amerindian ancestry. While socio-cultural and environmental factors were related to Amerindian ancestry, these factors did not account for the observed protective effects of Amerindian ancestry on asthma and rhinitis in these children. Our findings indicate that incorporation of genetic ancestry may further elucidate the disparities in the burden of asthma and rhinitis in the fast growing, diverse Hispanic population in the US.
