# Correction: Genetic Ancestry and Asthma and Rhinitis Occurrence in Hispanic Children: Findings from the Southern California Children's Health Study

**DOI:** 10.1371/journal.pone.0139397

**Published:** 2015-09-25

**Authors:** Muhammad T. Salam, Tigran Avoundjian, Wendy M. Knight, Frank D. Gilliland

There are errors in Table 4. Please see the corrected Table 4 here.

**Table 4:** Associations between Participants’ Sociodemographic Characteristics and Exposures with Amerindian Ancestry

	Categories based on proportions of Amerindian Ancestry				P-value^a^
	<0.25	≥0.25 and <0.50	≥0.50 and <0.75	≥ 0.75	
	(N = 311)	(N = 245)	(N = 409)	(N = 853)	
	N (%)	N (%)	N (%)	N (%)	
Boys	165 (53.05)	115 (46.94)	221 (54.03)	424 (49.71)	.24
Children with asthma	61 (20.61)	38 (16.67)	53 (13.52)	72 (8.97)	**<.001**
Children with rhinitis	136 (43.87)	104 (42.98)	138 (34.24)	252 (30.25)	**<.001**
Annual family income					
<$15,000	26 (9.00)	25 (11.68)	60 (17.19)	204 (29.96)	
$15,000–$49,999	74 (25.61)	67 (31.31)	174 (49.86)	374 (54.92)	**<.001**
≥$50,000	189 (65.40)	122 (57.01)	115 (32.95)	103 (15.12)	
Maternal birth place					
United States	273 (87.78)	179 (73.06)	198 (48.41)	208 (24.38)	
Mexico	16 (5.14)	53 (21.63)	200 (48.90)	600 (70.34)	**<.001**
Other	22 (7.07)	13 (5.31)	11 (2.69)	45 (5.28)	
Birth by Cesarean section	65 (20.90)	65 (26.53)	77 (18.83)	137 (16.06)	**.002**
Preterm birth	33 (10.65)	31 (12.81)	43 (10.72)	60 (7.30)	**.03**
Low birth weight (<2500g)	9 (2.89)	14 (5.71)	23 (5.62)	46 (5.39)	.29
Parent completed Spanish questionnaire	9 (2.89)	34 (13.88)	142 (34.72)	504 (59.09)	**<.001**
Child has health Insurance	288 (94.12)	219 (91.63)	350 (86.63)	672 (83.37)	**<.001**
Any parental history of asthma/allergy	180 (61.86)	123 (53.71)	151 (40.70)	205 (27.19)	**<.001**
Exposed to maternal smoking in utero	37 (12.05)	10 (4.22)	13 (3.31)	13 (1.57)	**<.001**
Exposed to paternal smoking in utero	64 (21.40)	33 (13.92)	53 (13.52)	78 (9.61)	**<.001**
Children exposed to second hand smoke	23 (7.44)	18 (7.47)	34 (8.56)	51 (6.20)	.49
Residential distance from a major road					
>300m	137 (48.41)	109 (48.66)	129 (34.31)	253 (34.24)	
151–300m	63 (22.26)	40 (17.86)	106 (28.19)	211 (28.55)	**<.001**
75–150m	45 (15.90)	43 (19.20)	70 (18.62)	144 (19.49)	
<75m	38 (13.43)	32 (14.29)	71 (18.88)	131 (17.73)	
Presence of cat at home	84 (27.18)	56 (23.43)	31 (7.89)	41 (5.09)	**<.001**
Presence of dog at home	112 (36.25)	72 (30.13)	88 (22.39)	161 (19.98)	**<.001**
Presence of cockroach at home	15 (5.00)	16 (7.05)	38 (10.19)	133 (18.12)	**<.001**
BMI Category					
Underweight	26 (9.12)	6 (2.59)	14 (3.69)	32 (4.08)	
Normal	182 (63.86)	158 (68.10)	242 (63.85)	448 (57.14)	**<.001**
Overweight	42 (14.74)	39 (16.81)	59 (15.57)	129 (16.45)	
Obese	35 (12.28)	29 (12.50)	64 (16.89)	175 (22.32)	

^a^ P values from Pearson chi-square tests for overall association with categories of Amerindian ancestry. Statistically significant P-values are in **bold**.
